# Elevated Serum Tenascin-C Predicts Mortality in Critically Ill Patients With Multiple Organ Dysfunction

**DOI:** 10.3389/fmed.2021.759273

**Published:** 2021-11-26

**Authors:** Yunyu Xu, Nanyang Li, Jiamin Gao, Da Shang, Min Zhang, Xiaoyi Mao, Ruiying Chen, Jianming Zheng, Ying Shan, Mingquan Chen, Qionghong Xie, Chuan-Ming Hao

**Affiliations:** ^1^Division of Nephrology, Huashan Hospital, Fudan University, Shanghai, China; ^2^Department of Emergency, Huashan Hospital, Fudan University, Shanghai, China; ^3^Department of Infectious Diseases, Huashan Hospital, Fudan University, Shanghai, China

**Keywords:** tenascin-C, critically ill patients, multiple organ dysfunction (MODS), mortality, biomarker

## Abstract

**Background:** Multiple organ dysfunction is a complex and lethal clinical feature with heterogeneous causes and is usually characterized by tissue injury of multiple organs. Tenascin-C (TNC) is a matricellular protein that is rarely expressed in most of the adult tissues, but re-induced following injury. This study aimed to evaluate serum TNC in predicting mortality in critically ill patients with multiple organ dysfunction.

**Methods:** Adult critically ill patients with at least two organs dysfunction and an increase of Sequential Organ Failure Assess (SOFA) score ≥ 2 points within 7 days were prospectively enrolled into two independent cohorts. The emergency (derivation) cohort was a consecutive series and the patients were from Emergency Department. The inpatient (validation) cohort was a convenience series and the patients were from medical wards. Their serum samples at the first 24 h after enrollment were collected and subjected to TNC measurement using ELISA. The association between serum TNC level and 28-day all-cause mortality was investigated, and then the predictive value of serum TNC was analyzed.

**Results:** A total of 110 patients with a median age of 64 years (53, 73) were enrolled in the emergency cohort. Compared to the survivors, serum TNC in the non-survivors was significantly higher (467.7 vs. 197.5 ng/ml, *p* < 0.001). Multivariate logistic regression analysis revealed that the association between serum TNC and 28-day mortality was independent of sepsis or critical illness scores such as SOFA, Acute Physiology and Chronic Health Evaluation (APACHE II), and Simplified Acute Physiology Score (SAPS II), respectively (*p* < 0.001 for each). The area under receiver operating characteristic curve of serum TNC for predicting mortality was 0.803 (0.717–0.888) (*p* < 0.001), similar with SOFA 0.808 (0.725–0.891), APACHE II 0.762 (0.667–0.857), and SAPS II 0.779 (0.685–0.872). The optimal cut-off value of serum TNC was 298.2 ng/ml. Kaplan–Meier analysis showed that the survival of patients with serum TNC ≥ 300 ng/ml was significantly worse than that of patients with serum TNC < 300 ng/ml. This result was validated in the inpatient cohort. The sensitivity and specificity of serum TNC ≥ 300 ng/ml for predicting mortality were 74.3 and 74.7% in the emergency cohort, and 63.0 and 70.1% in the inpatient cohort, respectively.

**Conclusion:** Serum TNC was associated with mortality in critically ill patients with multiple organ dysfunction, and would be used as a prognostic tool for predicting mortality in this population.

## Introduction

Multiple organ dysfunction, defined as more than one organ system deranged, is a complex and lethal clinical feature with highly heterogeneous causes and clinical manifestations. Different diseases, such as sepsis, malignant tumor, cardiovascular/cerebrovascular diseases, and surgery or trauma can lead to multiple organ dysfunction. The most common organs affected are kidneys, lungs, heart, hematologic system, liver, and central nervous system (1). Prognoses of critically ill patients with multiple organ dysfunction are usually poor. Therefore, accurate prediction of outcomes in these patients can guide physicians in their communication and decision making. However, the outcome of these patients at risk has many relative effects including age, gender, the severity of illness, comorbidities, diagnosis, and response to therapy, which makes the prediction of prognosis difficult and inaccurate. During the last three decades, several physiology-based ICU prognostic models have emerged. The main prognostic models for assessing the overall severity of illness in critically ill adults are Acute Physiology and Chronic Health Evaluation (APACHE), Simplified Acute Physiology Score (SAPS), and Mortality Prediction Model (MPM) (2). These models, as well as Sequential Organ Failure Assess (SOFA), which was primarily designed to describe the degree of organ dysfunction in critically ill patients (3), have been found to predict mortality effectively in different clinical conditions (4–6). However, limitations for these scoring systems do exist since they are all obtained by calculating a lot of components, which makes their clinical practice complicated. So, new effective outcome examination and guidance in critically ill patients with multiple organ dysfunction are strongly demanded.

Serum tenascin-C (TNC) has been reported to be significantly increased in critically ill patients and associated with the severity of diseases (7). TNC is a matricellular protein that is widely expressed during embryonic development and absent in most of the adult tissues, but re-induced following injury (8). TNC contains multiple functional domains that primarily regulate the interaction of cells with other extracellular matrix components and growth factors, thereby modulating cellular processes such as cell adhesion, proliferation, survival, migration, and differentiation (8, 9). Previous studies showed that TNC expression can be induced in different organs without disease specificity, such as heart tissue with myocarditis (10, 11), acute myocardial infarction (12), and dilated cardiopathy (10, 13), liver tissue with hepatitis (14), lung tissue with fibrosis (15, 16), and kidney tissue with glomerulonephritis and fibrosis (17, 18). The increased TNC in injured tissues can be released into a circulating system that results in serum TNC elevation. There were several studies, which revealed that serum TNC was associated with the mortality of different critical illnesses, such as sepsis, acute aortic dissection (AD), and myocarditis (7, 19, 20). However, the clinical significance of serum TNC levels in critically ill patients with multiple organ dysfunction remains uncertain.

Based on the previous studies, we hypothesized that serum TNC, representative of the quantity and severity of organ damage, was associated with mortality of critically ill patients with multiple organ dysfunction. In this prospective study, we detected serum TNC in critically ill patients with multiple organ dysfunction from two independent cohorts (emergency cohort and inpatient cohort) and aimed to evaluate the serum TNC. First, we investigated the association between serum TNC and mortality, and then examined the predictive performance of serum TNC for 28-day mortality in the emergency cohort and validated it in the inpatient cohort. We determined the cut-off value using the Youden index and evaluated the dichotomized predictive ability, and further combined the TNC value with critical scores to improve the predictive values. This work provided a strong basis for the adoption of serum TNC as an effective prognostic biomarker in critically ill patients with multiple organ dysfunction.

## Materials and Methods

### Study Design and Participants

The study was a prospective observational cohort with prespecified outcome and procurement of biological specimens. Other than blood draws, there were no study-related interventions, and all clinical care was at the discretion of the clinical teams caring for the study subjects. The study subjects were recruited in two separated cohorts, although they were from a single hospital—Huashan Hospital, Fudan University, which is a large size tertiary comprehensive hospital with more than 1,200 beds in the city of Shanghai, China. The emergency cohort, which was used as a derivation set, was a consecutive series and the subjects were recruited in Emergency Department from January 2018 to December 2019. Different from the emergency cohort, the inpatient cohort was a convenience series and the subjects were recruited in Divisions of Internal Department (medical wards) from January 2015 to December 2016. This cohort was used as a validation set. The study design was presented in Supplementary Material 1.

All the study subjects were adults and met the criteria of at least two organs dysfunction and acute organ injury with an increase of SOFA ≥ 2 points within 7 days caused by risk factors including infection, malignancy, rheumatic diseases, trauma, cardiovascular events, and others such as diabetic ketoacidosis and pancreatitis. Organ dysfunction was defined by reference to SOFA, which is composed of scores from six organ systems. Patients with (1) age <18 years; (2) died within 48 h after acute onset (e.g., trauma, acute myocardial infarction, and stroke); and (3) no information on follow-up outcome were excluded. Sepsis was defined as an acute change in total SOFA score ≥ 2 points consequent to the infection (21). These patients were first to our hospital or transferred from others and followed up prospectively from enrollment to death or 28 days. The primary endpoint was all-cause mortality. This study was approved by the ethics review board at the Huashan Hospital, Fudan University. The informed consent was signed by the patients or their authorized representatives.

### Blood Sample and TNC Measurement

Serum specimens in both inpatient and emergency cohorts were obtained within 24 h after study enrollment and stored at −80°C for analysis. TNC in serum was measured in 1:50 dilution using quantitative ELISA kits (IBL, Lot. 27767), according to the instructions of the manufacturer. TNC detected by this ELISA kit is the large molecular weight variant.

### Clinical Data

At enrollment, clinical characteristics of the patients including age, gender, comorbidities, and vital signs were recorded by the researchers. Laboratory measurements including blood routine, blood biochemistry, arterial blood gas analysis, coagulation markers, and D-dimer, lactic dehydrogenase (LDH) and C-reactive protein (CRP) were carried out within 24 h after enrollment. Major causes of acute organ dysfunction were determined by two independent physicians. The disease severity was assessed by critical illness scoring systems, including SOFA, APACHE II, and SAPS II. As mentioned above, SOFA was calculated by scores from six organ systems which were graded from 0 to 4 by the degree of dysfunction (3). APACHE II was calculated based on the worst values of 12 physiologic criteria [body temperature, mean arterial blood pressure, heart rate, respiratory rate, oxygenation, arterial PH, hematocrit, white blood cell count, serum levels of sodium, potassium, creatinine, and Glasgow Coma Scale (GCS)] during the first 24 h after enrollment, as well as age and previous health status (22). SAPS II was calculated by 17 variables including 12 physiologic factors (body temperature, mean arterial blood pressure, heart rate, urine output, oxygenation index, white blood cell count, arterial bicarbonate, serum levels of sodium, potassium, urea nitrogen, bilirubin, and GCS), as well as age, type of admission, and three variables regarding underlying diseases (23).

### Statistical Analysis

Continuous variables were presented as mean (SD) for normal distribution and median (IQR) for non-normal distribution. Categorical variables were presented as frequencies and percentages. Comparisons between two groups were performed using independent *t*-test or Mann–Whitney *U*-test for continuous variables and Pearson's Chi-square test or Fisher's exact test for categorical variables. The data were analyzed in the following steps. First, the association between serum TNC and 28-day all-cause mortality were analyzed and multivariable logistic regression was used to adjust the potential confounders including age, gender, and severity of the disease. The covariates sepsis, SOFA, APACHE II, or SAPS II, which were clinically associated with the survival were included in the multivariable logistic regression, respectively. Second, the predictive performance of serum TNC for 28-day mortality was evaluated by the area under the receiver operating characteristic (ROC) curve (AUC) in both cohorts. Third, the optimal cut-off value was determined by the Youden index. According to the optimal cut-off value of serum TNC in the emergency (derivation) cohort, the study population was divided into TNC ≥ 300 ng/ml and TNC < 300 ng/ml groups. Then the association between serum TNC and disease severity, as well as all-cause mortality were analyzed. Kaplan–Meier survival curves were drawn to evaluate the difference of mortality between serum TNC ≥ 300 ng/ml and <300 ng/ml groups, and the log-rank test was used for comparison. Finally, according to different serum TNC levels, the sensitivity and specificity were also calculated. These results were obtained from the emergency (derivation) cohort and validated in the inpatient (validation) cohort. All statistical analyses were performed with SPSS 24.0 and Graph-Pad Prism 7.0 with a statistical significance of *p*-value < 0.05.

## Results

### Cohort Characteristics

Baseline patient characteristics for the emergency and inpatient cohorts are shown in Table 1. A total of 110 critically ill patients with median (IQR) age of 64 years (53, 73) and 67% men were enrolled in the emergency cohort. The organ dysfunction defined as a SOFA score ≥ 1 included 60.0% in coagulation disorder, 50.9% in liver, 46.4% in respiration system, 43.6% in the neurological system, 38.2% in kidney, and 11.8% in cardiovascular systems. Among them, 58 (52.7%) had a history of chronic organ dysfunction. In the patients with chronic organ dysfunction, the predominant causes of acute organ injury were also acute exacerbation of chronic disease (63.8%) and infection (89.7%). In the patients without chronic organ dysfunction, the predominant causes of acute organ injury were also infection (86.5%) and subsequent metabolic diseases (19.2%), malignancy (17.3%), and activity of connective tissue disease (13.5%). Of the 110 patients, 36.4% had acute kidney injury, 55.5% had sepsis, and 16.4% were ventilation dependent at the enrollment, and 31.8% died during the follow-up.

**Table 1:** Clinical characteristics of patients with multiple organ dysfunction.

	**Emergency (derivation) cohort**				**Inpatient (validation) cohort**			
	**Total**	**Survivor**	**Non-survivor**	***P***	**Total**	**Survivor**	**Non-survivor**	***P***
	***n* = 110**	***n* = 75**	***n* = 35**		***n* = 115**	***n* = 88**	***n* = 27**	
**Age (years)**	64 (53, 73)	63 (52, 72)	65 (54, 73)	0.672	56 (38, 66)	53 (36, 65)	62 (50, 73)	0.054
**Male**, ***n*** **(%)**	74 (67.3%)	51 (68.0%)	23 (65.7%)	0.813	75 (65.2%)	59 (67.0%)	16 (59.3%)	0.459
**Laboratory**								
Creatinine (mg/dL)	1.0 (0.6, 1.9)	78.5 (55.5, 143.5)	109.0 (57.0, 272.0)	0.249	3.0 (0.9, 6.2)	271.5 (79.3, 672.5)	231.0 (80.0, 383.0)	0.229
LDH (U/L)	297.0 (222.0, 595.0)	286.0 (209.8, 594.0)	419.0 (245.0, 626.0)	0.283	244.0 (196.0, 363.0)	237.0 (186.0, 360.0)	327.5 (221.3, 1,091.5)	0.080
CRP (mg/L)	68.7 (17.3, 185.8)	78.2 (13.8, 190.3)	39.5 (23.5, 178.3)	0.642	16.8 (5.5, 78.7)	13.5 (3.3, 73.6)	53.0 (16.8, 92.8)	0.063
Platelet (×10^9^/L)	116.0 (54.3, 214.0)	139.0 (78.0, 220.0)	70.0 (46.0, 173.0)	0.019	115.0 (72.5, 227.0)	171.0 (104.0, 239.0)	62.0 (32.0, 155.5)	<0.001
INR	1.2 (1.1, 1.6)	1.2 (1.1, 1.4)	1.4 (1.1, 1.9)	0.013	1.1 (1.0, 1.4)	1.1 (1.0, 1.2)	1.4 (1.2, 1.9)	<0.001
D-dimer (μg/mL)	3.8 (1.9, 7.8)	3.4 (1.5, 6.5)	4.3 (2.3, 9.8)	0.116	2.3 (1.0, 6.7)	1.8 (0.5, 4.0)	5.3 (2.5, 13.0)	<0.001
Total Bilirubin (mg/dL)	1.2 (0.6, 3.5)	20.3 (8.6, 59.3)	26.5 (11.1, 75.4)	0.194	0.6 (0.3, 1.4)	8.1 (4.6, 16.3)	20.9 (11.0, 65.0)	<0.001
**Involved organs**								
Cardiovascular dysfunction	13 (11.8%)	9 (12.0%)	4 (11.4%)	0.931	15 (13.0%)	9 (10.2%)	6 (22.2%)	0.107
Respiratory dysfunction	51 (46.4%)	30 (40.0%)	21 (60.0%)	0.051	41 (35.7%)	27 (30.7%)	14 (51.9%)	0.045
Renal dysfunction	42 (38.2%)	25 (33.3%)	17 (48.6%)	0.127	79 (68.7%)	60 (68.2%)	19 (70.4%)	0.831
Hepatic dysfunction	56 (50.9%)	37 (49.3%)	19 (54.3%)	0.630	33 (28.7%)	19 (21.6%)	14 (51.9%)	0.002
Neurological dysfunction	48 (43.6%)	28 (37.3%)	20 (57.1%)	0.052	25 (21.7%)	13 (14.8%)	12 (44.4%)	0.001
Coagulation disorder	66 (60.0%)	41 (54.7%)	25 (71.4%)	0.096	52 (45.2%)	33 (37.5%)	19 (70.4%)	0.003
**Chronic organ dysfunction**	58 (52.7%)	37 (49.3%)	21 (60.0%)	0.227	78 (67.8%)	58 (65.9%)	20 (74.1%)	0.429
**Causes of acute organ injury in patients with chronic organ dysfunction**								
Acute exacerbation of chronic disease	37 (63.8%)	23 (30.7%)	14 (40.0%)	0.337	62 (79.5%)	48 (54.5%)	14 (51.9%)	0.807
Infection	52 (89.7%)	31 (41.3%)	21 (60.0%)	0.069	36 (46.2%)	21 (23.9%)	15 (55.6%)	0.002
Other	19 (32.8%)	12 (16.0%)	7 (20.0%)	0.607	33 (42.3%)	29 (33.0%)	4 (14.8%)	0.070
**Causes of acute organ injury in patients without chronic organ dysfunction**								
Infection	45 (86.5%)	32 (42.7%)	13 (37.1%)	0.585	35 (94.6%)	25 (28.5%)	10 (37.0%)	0.996
Malignancy	9 (17.3%)	6 (8.0%)	3 (8.6%)	0.919	6 (16.2%)	2 (2.3%)	4 (14.8%)	0.011
Activity of connective tissue disease	7 (13.5%)	5 (6.7%)	2 (5.7%)	0.850	5 (13.5%)	5 (5.7%)	0	0.207
Metabolic disease (e.g., DKA)	10 (19.2%)	8 (10.7%)	2 (5.7%)	0.402	3 (8.1%)	2 (2.3%)	1 (3.7%)	0.685
Surgery/Trauma	5 (9.6%)	5 (6.7%)	0	0.120	2 (5.4%)	2 (2.3%)	0	0.431
Cardiovascular/Cerebrovascular events	4 (7.7%)	2 (2.7%)	2 (5.7%)	0.429	8 (21.6%)	6 (6.8%)	2 (7.4%)	0.917
Other	15 (28.8%)	10 (13.3%)	5 (14.3%)	0.893	9 (24.3%)	9 (10.2%)	0	0.085
**Sepsis**, ***n*** **(%)**	61 (55.5%)	35 (46.7%)	26 (74.3%)	0.007	31 (27.0%)	18 (20.5%)	13 (48.1%)	0.005
**Critical illness score**								
SOFA	6.0 (3.0, 8.0)	4.0 (3.0, 7.0)	8.0 (7.0, 10.0)	<0.001	6.0 (4.0, 9.0)	5.0 (3.0, 8.0)	8.5 (6.0, 12.0)	<0.001
APACHE II	16.0 (11.0, 21.0)	14.0 (10.0, 18.0)	20.0 (15.0, 25.0)	<0.001	14.0 (10.0, 20.0)	13.5 (8.3, 17.0)	19.0 (14.0, 23.0)	<0.001
SAPS II	43.0 (35.0, 54.0)	39.0 (32.0, 47.0)	55.0 (43.0, 65.0)	<0.001	37.0 (30.0, 43.0)	33.5 (26.3, 40.5)	44.0 (41.0, 59.0)	<0.001
**TNC**	229.4 (141.6, 472.5)	197.5 (97.7, 343.8)	467.0 (267.4, 786.3)	<0.001	210.2 (96.8, 469.6)	202.6 (91.4, 324.9)	584.4 (164.4, 902.6)	0.002

*TNC, tenascin-C; LDH, lactate dehydrogenase; CRP, C-reactive protein; INR, International Normalized Ratio; DKA, diabetic ketoacidosis; SOFA, Sequential Organ Failure Assessment; APACHE II, Acute Physiology and Chronic Health Evaluation II; SAPS II, Simplified Acute Physiology Score II*.

A total of 115 critically ill patients with median (IQR) age of 56 years (38, 66) and 65.2% men were enrolled in the inpatient cohort. Seventy percent were from the Division of Nephrology or had nephrology consultation. The organ dysfunction, with the same inclusion criteria, included 68.7% in kidney, 45.2% in coagulation, 35.7% in respiration, 28.7% in liver, 21.7% in the neurological system, and 13.0% in cardiovascular systems. Among them, 78 (67.8%) had a history of chronic organ dysfunction. In the patients with chronic organ dysfunction, the predominant causes of acute organ injury were acute exacerbation of chronic disease (79.5%) and infection (46.2%). In the patients without chronic organ dysfunction, the predominant causes of acute organ injury were infection (94.6%), and subsequent cardiovascular/cerebrovascular events (21.6%), malignancy (16.2%), and the activity of connective tissue disease (13.5%). Of the 115 patients, 45.2% had acute kidney injury, 31% had sepsis and 7% were ventilation dependent at the enrollment, and 23.5% died during the follow-up.

### Serum TNC Was Significantly Higher in the Patients Who Died Within 28 Days

In the emergency cohort, serum TNC in the non-survivors was 467.7 ng/ml, significantly higher than that of 197.5 ng/ml in the survivors (*p* < 0.001) (Table 1; Supplementary Material 2). The severity was even more in non-survivors with significantly higher critical illness scores, including SOFA (8.0 vs. 4.0), APACHE II (20.0 vs. 14.0), and SAPS II (55.0 vs. 39.0), and had a higher rate of sepsis (74.3 vs. 46.7%, *p* = 0.007) than the survivors (*p* < 0.001 for all). They also had significantly lower blood platelet levels and higher internal normalized ratio (INR). For age, gender, involved organs, and causes of acute organ injury, there was no significant difference between the survivors and non-survivors (Table 1). Multivariate logistic regression analysis revealed that the association between serum TNC [adjusted OR (95% CI), 1.656 (1.288, 2.130)] and 28-day mortality was independent of sepsis [adjusted OR (95% CI), 1.255 (0.433, 3.635)] or critical illness scores such as SOFA [adjusted OR (95% CI), 1.423 (1.181, 1.715)], APACHE II [adjusted OR (95% CI), 1.142 (1.053, 1.239)], and SAPS II [adjusted OR(95% CI), 1.076 (1.031, 1.123)], respectively (*p* < 0.001 for all) (Table 2).

**Table 2:** Multivariate analysis for risk factors of all-cause mortality in patients with multiple organ dysfunction.

**Variables**	**Emergency (derivation) cohort**		**Inpatient (validation) cohort**	
	**Adjusted OR (95% CI)**	***p*-value**	**Adjusted OR (95% CI)**	***p*-value**
**Model 1**				
Age	0.980 (0.950, 1.010)	0.191	1.012 (0.985, 1.040)	0.377
Gender	1.341 (0.474, 3.794)	0.581	0.604 (0.219, 1.668)	0.331
Sepsis	1.255 (0.433, 3.635)	0.676	1.718 (0.576, 5.121)	0.332
TNC (per 100 ng/ml increase)	1.656 (1.288, 2.130)	<0.001	1.261 (1.084, 1.467)	**0.004**
**Model 2**				
Age	0.979 (0.946, 1.013)	0.215	1.014 (0.986, 1.042)	0.341
Gender	1.475 (0.469, 4.634)	0.505	0.651 (0.214, 1.978)	0.449
SOFA	1.423 (1.181, 1.715)	<0.001	1.275 (1.088, 1.495)	0.003
TNC (per 100 ng/ml increase)	1.678 (1.276, 2.206)	<0.001	1.137 (1.024, 1.262)	**0.016**
**Model 3**				
Age	0.976 (0.945, 1.009)	0.150	1.007 (0.980, 1.035)	0.629
Gender	1.516 (0.496, 4.638)	0.466	0.761 (0.262, 2.210)	0.616
APACHE II	1.142 (1.053, 1.239)	0.001	1.138 (1.034, 1.253)	0.008
TNC (per 100 ng/ml increase)	1.630 (1.276, 2.083)	<0.001	1.118 (1.011, 1.237)	0.030
**Model 4**				
Age	0.969 (0.936, 1.002)	0.558	0.993 (0.962, 1.025)	0.663
Gender	1.429 (0.468, 4.363)	0.556	0.653 (0.217, 1.964)	0.449
SAPS II	1.076 (1.031, 1.123)	0.001	1.092 (1.032, 1.157)	0.002
TNC (per 100 ng/ml increase)	1.524 (1.203, 1.930)	<0.001	1.109 (0.997, 1.234)	0.057

*TNC, tenascin-C; SOFA, Sequential Organ Failure Assessment; APACHE II, Acute Physiology and Chronic Health Evaluation II; SAPS II, Simplified Acute Physiology Score II*.

In the inpatient cohort, serum TNC had a similar direction and magnitude (584.4 ng/ml in the non-survivors vs. 202.6 ng/ml in the survivors, *p* = 0.002) with that in the emergency cohort (Table 1; Supplementary Material 2). The severity was more in non-survivors and had more sepsis, higher INR, and lower blood platelet levels (Table 1). However, for involved organs, the non-survivors had more lung, liver, brain, and coagulation system involved than the survivors, and the cause of acute organ injury was more infection and malignant diseases (Table 1). Multivariate logistic regression analysis revealed that the association between serum TNC [adjusted OR (95% CI), 1.261 (1.084, 1.467)] and 28-day mortality was independent of sepsis [adjusted OR (95% CI), 1.718 (0.576, 5.121), *p* = 0.004] or critical illness scores such as SOFA [adjusted OR (95% CI), 1.275 (1.088, 1.495), *p* = 0.016] and APACHE II [adjusted OR (95% CI), 1.138 (1.034, 1.253), *p* = 0.030] respectively, but not SAPS II [adjusted OR (95% CI), 1.092 (1.032, 1.157), *p* = 0.057] (Table 2).

### The Performance of Serum TNC for Predicting Mortality in Critically Ill Patients

**Figure 1:**
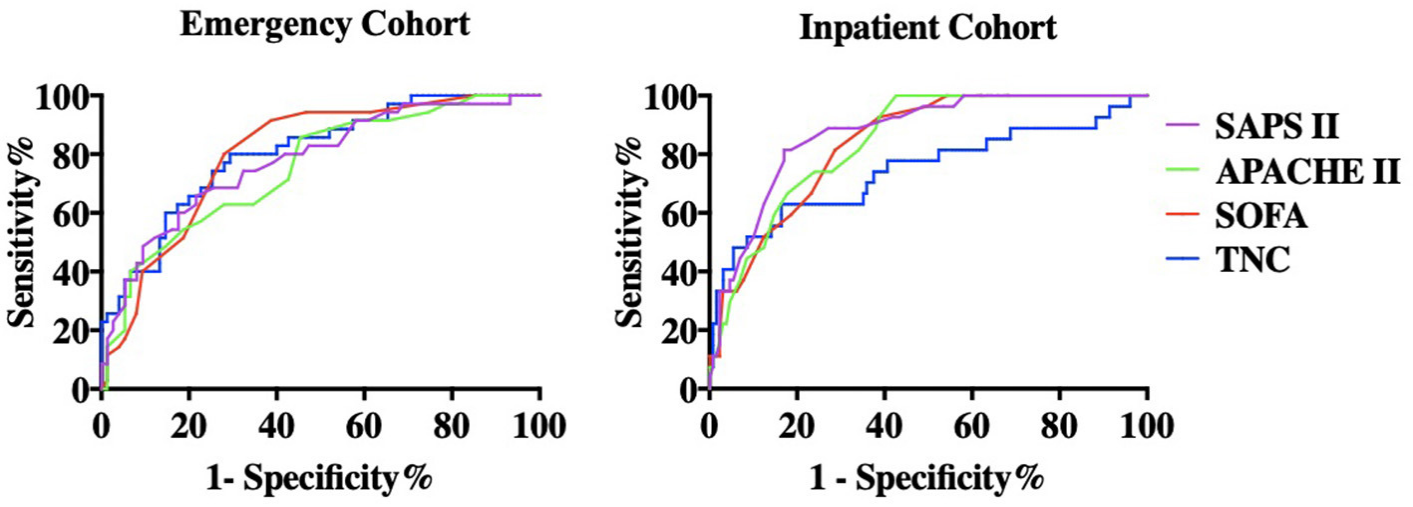
Receiver Operating Characteristics (ROC) curve in both cohorts. The areas under ROC (AUC) of serum TNC, SOFA, APACHE II, and SAPS II for predicting all-cause mortality were 0.803, 0.808, 0.762, and 0.779 (*p* < 0.001 for all) in the emergency (derivation) cohort and 0.745, 0.844, 0.846, and 0.872 (*p* < 0.001) in the inpatient (validation) cohort. In the emergency cohort, there was no significant difference between serum TNC and the three critical illness scores. In the inpatient cohort, the AUC of serum TNC was significantly lower than SAPS II (*p* = 0.032) while there was no significant difference between serum TNC and SOFA or APACHE II.

In the emergency cohort, the AUCs of serum TNC, SOFA, APACHE II, and SAPS II for predicting 28-day mortality were 0.803 (0.717–0.888), 0.808 (0.725–0.891), 0.762 (0.667–0.857), and 0.779 (0.685–0.872), respectively (*p* < 0.001 for all). There was no statistically significant difference in AUCs between serum TNC and the three critical illness scores (Supplementary Material 3; Figure 1). The optimal cut-off value of serum TNC calculated by the Youden index was 298.2 ng/ml. In the inpatient cohort, the AUCs of serum TNC, SOFA, APACHE II, and SAPS II for predicting 28-day mortality were 0.745 (0.624–0.865), 0.844 (0.776–0.912), 0.846 (0.780–0.912), and 0.872 (0.808–0.936), respectively (*p* < 0.001 for all). The ROC of serum TNC was lower than SAPS II (*p* = 0.032), whereas there was no significant difference between serum TNC and SOFA or APACHE II (Supplementary Material 3; Figure 1).

**Figure 2:**
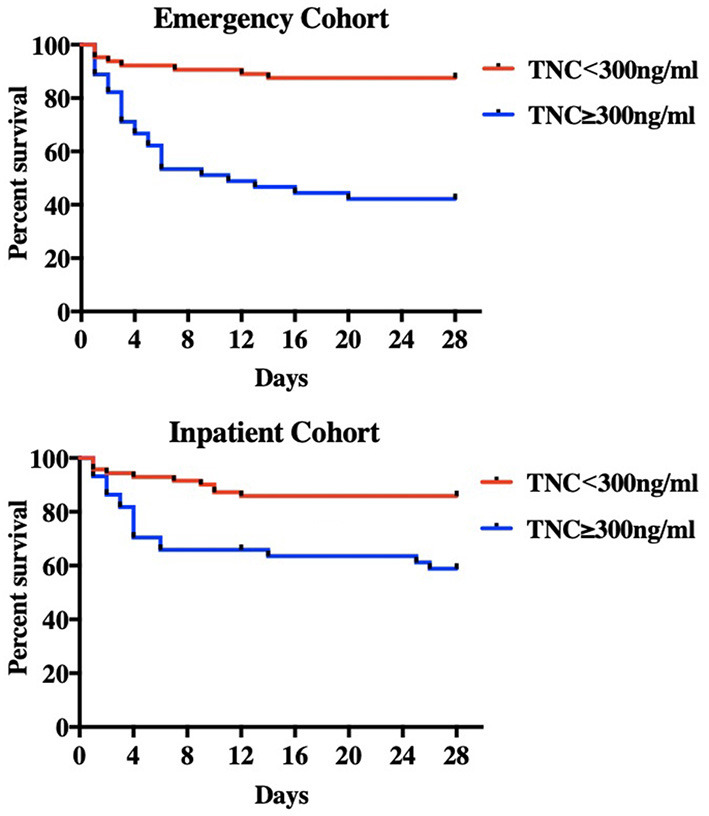
Kaplan-Meier survival curve. By reference to the optimal cut-off value, the patients were divided into serum TNC ≥ 300 ng/ml and TNC < 300 ng/ml groups. Compared with lower TNC group, the higher TNC group had a significantly lower survival rate in both emergency (derivation) cohort (log rank test, *p* < 0.0001) and inpatient (validation) cohort (log rank test, *p* = 0.002).

According to the optimal cut-off value, the study population was divided into TNC ≥ 300 ng/ml and TNC <300 ng/ml groups. Compared to the patients with lower TNC, patients with higher TNC were older and more severe with significantly higher critical illness scores including SOFA, APACHE II, and SAPS II (*p* < 0.01 for all), and had a significantly higher 28-day mortality (57.8 vs. 13.8%, *p* < 0.001). This result was validated in the inpatient cohort, which showed that the mortality was 38.6% in the non-survivors and 14.1% in the survivors (*p* = 0.003) (Table 3). Kaplan–Meier analysis showed that the survival of patients with serum TNC ≥ 300 ng/ml was significantly worse than that of patients with serum TNC <300 ng/ml in both cohorts (log-rank test, *p* < 0.001 in the emergency cohort and *p* = 0.002 in the inpatient cohort) (Figure 2). As a single biomarker, the sensitivity and specificity of serum TNC ≥ 300 ng/ml for predicting mortality was 74.3 and 74.7% in the emergency cohort, while they were 63.0 and 70.1% in the inpatient cohort, respectively. If TNC ≥ 450 ng/ml, the specificity was 85.3% in the emergency cohort and 79.3% in the inpatient cohort (Table 4).

**Table 3:** Comparisons between patients with serum TNC ≥ 300 ng/ml and serum TNC < 300 ng/ml.

	**Emergency (derivation) cohort**			**Inpatient (validation) cohort**		
	**TNC < 300 ng/ml**	**TNC ≥ 300 ng/ml**	***P***	**TNC < 300 ng/ml**	**TNC ≥ 300 ng/ml**	***P***
	***n* = 65**	***n* = 45**		***n* = 71**	***n* = 44**	
Age (years)	61 (48, 71)	67 (60, 78)	0.002	51 (35, 62)	64 (49, 74)	0.001
Male, *n* (%)	46 (70.8%)	28 (62.2%)	0.350	42 (59.2%)	33 (75.0%)	0.084
Creatinine (mg/dL)	0.8 (0.6, 1.5)	1.3 (0.7, 3.2)	0.043	1.9 (0.7, 5.0)	4.5 (2.4, 7.8)	0.001
CRP (mg/L)	42 (7, 169)	135 (32, 200)	0.023	11 (3, 23)	81 (10, 135)	0.002
D-dimer (μg/mL)	3.4 (1.4, 4.6)	5.6 (2.6, 11.1)	0.002	1.5 (0.5, 2.8)	4.6 (2.3, 12.6)	<0.001
Sepsis, *n* (%)	25 (38.5%)	36 (80.0%)	<0.001	11 (15.5%)	20 (45.5%)	<0.001
SOFA	4.0 (3.0, 7.5)	7.0 (6.0, 8.5)	0.003	5.0 (3.0, 8.0)	7.0 (5.0, 9.0)	0.001
APACHE II	14.0 (10.0, 18.0)	18.0 (14.0, 22.5)	0.003	11.0 (7.0, 16.0)	17.0 (15.0, 22.0)	<0.001
SAPS II	38.0 (31.0, 49.8)	47.0 (41.5, 62.0)	<0.001	33.0 (26.0, 41.0)	41.5 (35.5, 53.0)	<0.001
Mortality, *n* (%)	9 (13.8%)	26 (57.8%)	<0.001	10 (14.1%)	17 (38.6%)	0.003

*TNC, tenascin-C; CRP, C-reactive protein; SOFA, Sequential Organ Failure Assessment; APACHE II, Acute Physiology and Chronic Health Evaluation II; SAPS II, Simplified Acute Physiology Score II*.

**Table 4:** The diagnostic sensitivity and specificity of serum TNC and critical illness scores in emergency (derivation) and inpatient (validation) cohorts.

	**Emergency (derivation) cohort**			**Inpatient (validation) cohort**		
	***N***	**Sensitivity**	**Specificity**	***N***	**Sensitivity**	**Specificity**
TNC≥200 ng/ml	67	85.7%	50.7%	63	66.7%	48.2%
TNC≥300 ng/ml	45	74.3%	74.7%	63	66.7%	48.2%
TNC≥450 ng/ml	29	51.4%	85.3%	31	51.9%	79.3%

## Discussion

In this prospective study, we found that serum TNC was significantly higher in the critically ill patients with multiple organ dysfunction who died within 28 days in two independent cohorts. We revealed that the AUC of serum TNC for predicting mortality was similar to SOFA, APACHE II, and SAPS II. Kaplan–Meier analysis showed that the survival of patients with higher serum TNC was significantly worse than that of patients with lower serum TNC in both cohorts. These findings suggested that serum TNC was a useful prognostic tool for predicting 28-day mortality in critically ill patients with multiple organ dysfunction.

Previous studies had developed several models to predict the prognosis of critically ill patients. Among them, the APACHE II score and SAPS II, which were initially developed to predict hospital mortality in general ICU patients, have good prognostic performance (22, 23). However, they are calculated using the worst values from data collected in the first 24 h after ICU admission and are therefore not immediately available at the time of admission. They use 14 or 17 variables, also making their clinical practice not convenient. SOFA, another confirmed useful predictive model of critical illness, which is much simpler than APACHE II score and SAPS II, also measures six variables (4–6). Even so, the discrimination of these models remains unsatisfactory with AUCs varied from 0.7 to 0.9 in previous studies (24). It has been also reported that C-reactive protein (CRP), procalcitonin (PCT), lactate, and the markers of platelet function (such as thrombocytopenia and impaired platelet aggregation) were associated with the severity and mortality of multiple organ dysfunction (25–27). However, these biomarkers were more relevant to inflammation and infection and only showed limited value in predicting mortality in sepsis. Differently, serum TNC was upregulated in response to tissue injury regardless of causes and could be used to predict mortality in multiple organ dysfunction patients with the AUC as high as SOFA. As a single biomarker, it was convenient in clinical application.

The elevation of serum TNC may be explained by the upregulation of TNC in the injured tissues. It was postulated that persistent TNC expression was caused by prolonged inflammation and tissue injury. For example, intense TNC expression was observed at the site of infarction or active inflammation of the heart during the active phase but not in scar tissue during the healing phase (10, 11, 13, 28). Increased TNC expression was also found in lungs with progressive idiopathic pulmonary fibrosis (16), and in synovium with arthritis such as rheumatoid arthritis (29). Basic studies revealed that TNC could be induced by inflammatory and growth factors, oxidative stress, and hypoxia (30). Therefore, the increased TNC expression may reflect disease activity and progression of various diseases without organ specificity. This was the rationale we included patients with acute organ injury by an increase of SOFA ≥ 2 points within 7 days.

Consistent with TNC in tissues, serum TNC was also associated with the activity of various diseases (31–33). Page et al. reported that serum TNC, in rheumatoid arthritis, was associated with ultrasound-determined erosion scores and was decreased after treatment with infliximab and methotrexate (31). Zavada et al. found that serum TNC was positively associated with the disease activity score (SLEDAI) in SLE patients (34). Serum TNC was also associated with the severity and prognosis of various diseases (35–38). For example, serum TNC was correlated with the total occlusion and inflammation in myocardial infarction (39), and levels on day 5 after admission was an independent predictor for cardiac events during the follow-up period (24 ± 13 months) (40). In patients with heart failure, serum TNC was also positively correlated with the severity of left ventricular dysfunction and was an independent predictor for 12-month major adverse cardiac events (37). In patients with acute AD, serum TNC was a valuable biomarker for predicting in-hospital deaths (38). Acute, active, and systemic injury is a common feature of critically ill patients with multiple organ dysfunction, and serum TNC is probably significantly increased. But now, only a few studies focus on the predictive value of serum TNC for mortality in these patients. Meijer et al. examined plasma TNC during sepsis and non-septic critical illness and found that plasma TNC was reflective of disease severity more than an independent predictor of mortality (7). However, serum TNC, in our study, was not only positively associated with the critical illness scores such as SOFA, APACHE II, and SAPS II, but also independently associated with mortality after adjusting for these scores. This result was also supported by another study that focused on patients with sepsis and showed that serum TNC was positively correlated with SOFA scores and associated with 30-day mortality (41).

This study has several limitations. First, serum TNC in different stages may show different clinical significance. For example, higher serum TNC on admission predicted more hospitalization deaths in patients with acute AD (19), whereas a higher serum TNC on hospital day 7 predicted a lower risk of enlargement of the aortic lesion during the chronic stage (42). However, our study measured serum TNC one time in the acute stage. So, further studies with dynamic changes of serum TNC or in the chronic stage are required in the future. Second, the clinical utility of serum TNC may lie in the low negative predictive value, especially in patients with acute cardiovascular events. For example, patients with acute myocardial infarction or acute cerebral hemorrhage will probably die very soon after the events, but their basic serum TNC will not be as high as the patients with multiple organs injury, because the injured tissues may be limited to heart or brain in the early stage. By contrast, serum TNC is relatively useful for the assessment of illness severity in patients with multiple organ dysfunction. Third, the inpatient cohort enrolled the patients from different departments but not consecutive patients in ICUs. Selective bias included that more patients with kidney injury were enrolled than with other organs involved. The imbalance of patient enrollment influenced the interpreter of the results. However, the emergency cohort enrolling patients from the emergency department did not have this limitation. Fourth, there was no specific definition for critical illness, which also led to the various types of mortalities in different studies on this population. The inclusion criteria of our study were the patients with at least two organ dysfunction, which might be different from the traditional critically ill patients in the ICUs. Fifthly, TNC has multiple protein isoforms and which isoform has the strongest relationship with the outcome remains unknown. The ELISA kit used in this study measured the large TNC variant which was characteristic for some tumors. Finally, the low sample size also influenced the power of this study. So, larger, independent validation studies with consecutive patients in ICUs are needed to further support the utility of serum TNC in critically ill patients.

In summary, serum TNC, representative of tissue injury, is a novel, promising predictive marker for mortality in critically ill patients with multiple organ dysfunction. Incorporation of serum TNC into clinical practice and future investigation may bring better understanding and management in critically ill patients.

## Conclusions

Serum TNC was positively associated with the severity of illness and mortality, and could be used as a prognostic tool for predicting mortality in critically ill patients with multiple organ dysfunction.

## Data Availability Statement

The raw data supporting the conclusions of this article will be made available by the authors, without undue reservation.

## Ethics Statement

The studies involving human participants were reviewed and approved by the Ethics Review Board at Huashan Hospital, Fudan University. The patients/participants provided their written informed consent to participate in this study.

## Author Contributions

YX collected, analyzed, interpreted data, and drafted the manuscript. NL and JG collected and analyzed the data. DS and YS collected the data. QX and MC conceived the study, participated in its design and coordination, analyzed and interpreted data, drafted the manuscript, had full access to all the study data, and assume responsibility for the integrity of the data and the accuracy of the analysis. C-MH conceived the study, participated in its design, and helped to draft the manuscript. All authors approved the final manuscript.

## Funding

The study was supported by the National Natural Science Foundation of China Grants 81520108006, 81930120, and 81700673.

## Conflict of Interest

The authors declare that the research was conducted in the absence of any commercial or financial relationships that could be construed as a potential conflict of interest.

## Publisher's Note

All claims expressed in this article are solely those of the authors and do not necessarily represent those of their affiliated organizations, or those of the publisher, the editors and the reviewers. Any product that may be evaluated in this article, or claim that may be made by its manufacturer, is not guaranteed or endorsed by the publisher.

## References

[1] Proulx F Fayon M Farrell CA Lacroix J Gauthier M. Epidemiology of sepsis and multiple organ dysfunction syndrome in children. Chest. (1996) 109:1033–7. 10.1378/chest.109.4.1033 8635327

[2] Strand K Flaatten H. Severity scoring in the ICU: a review. Acta Anaesthesiol Scand. (2008) 52:467–78. 10.1111/j.1399-6576.2008.01586.x 18339152

[3] Vincent JL de Mendonca A Cantraine F Moreno R Takala J Suter PM. Use of the SOFA score to assess the incidence of organ dysfunction/failure in intensive care units: results of a multicenter, prospective study. Working group on “sepsis-related problems” of the European Society of Intensive Care Medicine. Crit Care Med. (1998) 26:1793–800. 10.1097/00003246-199811000-00016 9824069

[4] Raith EP Udy AA Bailey M McGloughlin S MacIsaac C Bellomo R. Prognostic Accuracy of the SOFA Score, SIRS Criteria, and qSOFA Score for In-Hospital Mortality Among Adults With Suspected Infection Admitted to the Intensive Care Unit. J Am Med Assoc. (2017) 317:290–300. 10.1001/jama.2016.20328 28114553

[5] Zhang Y Luo H Wang H Zheng Z Ooi OC. Validation of prognostic accuracy of the SOFA score, SIRS criteria, and qSOFA score for in-hospital mortality among cardiac-, thoracic-, and vascular-surgery patients admitted to a cardiothoracic intensive care unit. J Card Surg. (2020) 35:118–27. 10.1111/jocs.14331 31710762

[6] Brusca RM Simpson CE Sahetya SK Noorain Z Tanykonda V Stephens RS. Performance of critical care outcome prediction models in an intermediate care unit. J Intensive Care Med. (2019) 2019:885066619882675. 10.1177/0885066619882675 31635507 PMC8262077

[7] Meijer MT Uhel F Cremer OL Schultz MJ van der Poll T Consortium M. Tenascin C plasma levels in critically ill patients with or without sepsis: a multicentre observational study. Shock. (2019) 54:62–9. 10.1097/SHK.0000000000001481 31764620

[8] Midwood KS Hussenet T Langlois B Orend G. Advances in tenascin-C biology. Cell Mol Life Sci. (2011) 68:3175–99. 10.1007/s00018-011-0783-6 21818551 PMC3173650

[9] De Laporte L Rice JJ Tortelli F Hubbell JA. Tenascin C promiscuously binds growth factors *via* its fifth fibronectin type III-like domain. PLoS ONE. (2013) 8:e62076. 10.1371/journal.pone.0062076 23637968 PMC3630135

[10] Tsukada B Terasaki F Shimomura H Otsuka K Otsuka K Katashima T. High prevalence of chronic myocarditis in dilated cardiomyopathy referred for left ventriculoplasty: expression of tenascin C as a possible marker for inflammation. Hum Pathol. (2009) 40:1015–22. 10.1016/j.humpath.2008.12.017 19297005

[11] Morimoto S Imanaka-Yoshida K Hiramitsu S Kato S Ohtsuki M Uemura A. Diagnostic utility of tenascin-C for evaluation of the activity of human acute myocarditis. J Pathol. (2005) 205:460–7. 10.1002/path.1730 15685595

[12] Willems IE Arends JW Daemen MJ. Tenascin and fibronectin expression in healing human myocardial scars. J Pathol. (1996) 179:321–5. 10.1002/(SICI)1096-9896(199607)179:3<321::AID-PATH555>3.0.CO;2-8 8774490

[13] Yokokawa T Sugano Y Nakayama T Nagai T Matsuyama TA Ohta-Ogo K. Significance of myocardial tenascin-C expression in left ventricular remodelling and long-term outcome in patients with dilated cardiomyopathy. Eur J Heart Fail. (2016) 18:375–85. 10.1002/ejhf.464 26763891 PMC5066704

[14] El-Karef A Yoshida T Gabazza EC Nishioka T Inada H Sakakura T. Deficiency of tenascin-C attenuates liver fibrosis in immune-mediated chronic hepatitis in mice. J Pathol. (2007) 211:86–94. 10.1002/path.2099 17121418

[15] Carey WA Taylor GD Dean WB Bristow JD. Tenascin-C deficiency attenuates TGF-ss-mediated fibrosis following murine lung injury. Am J Physiol Lung Cell Mol Physiol. (2010) 299:L785–93. 10.1152/ajplung.00385.2009 20833777 PMC3006262

[16] Estany S Vicens-Zygmunt V Llatjos R Montes A Penin R Escobar I. Lung fibrotic tenascin-C upregulation is associated with other extracellular matrix proteins and induced by TGFbeta1. BMC Pulm Med. (2014) 14:120. 10.1186/1471-2466-14-120 25064447 PMC4123829

[17] Matsumoto K Hiraiwa N Yoshiki A Ohnishi M Kusakabe M. Tenascin-C expression and splice variant in habu snake venom-induced glomerulonephritis. Exp Mol Pathol. (2002) 72:186–95. 10.1006/exmp.2002.2432 12009782

[18] Fu H Tian Y Zhou L Zhou D Tan RJ Stolz DB. Tenascin-C is a major component of the fibrogenic niche in kidney fibrosis. J Am Soc Nephrol. (2017) 28:785–801. 10.1681/ASN.2016020165 27612995 PMC5328156

[19] Nozato T Sato A Hirose S Hikita H Takahashi A Endo H. Preliminary study of serum tenascin-C levels as a diagnostic or prognostic biomarker of type B acute aortic dissection. Int J Cardiol. (2013) 168:4267–9. 10.1016/j.ijcard.2013.04.211 23742930

[20] Imanaka-Yoshida K Tawara I Yoshida T. Tenascin-C in cardiac disease: a sophisticated controller of inflammation, repair, and fibrosis. Am J Physiol Cell Physiol. (2020) 319:C781–96. 10.1152/ajpcell.00353.2020 32845719

[21] Singer M Deutschman CS Seymour CW Shankar-Hari M Annane D Bauer M. The third international consensus definitions for sepsis and septic shock (sepsis-3). J Am Med Assoc. (2016) 315:801–10. 10.1001/jama.2016.0287 26903338 PMC4968574

[22] Knaus WA Draper EA Wagner DP Zimmerman JE. APACHE II a severity of disease classification system. Crit Care Med. (1985) 13:818–29. 10.1097/00003246-198510000-00009 3928249

[23] Le Gall JR Lemeshow S Saulnier F. A new Simplified Acute Physiology Score (SAPS II) based on a European/North American multicenter study. J Am Med Assoc. (1993) 270:2957–63. 10.1001/jama.270.24.2957 8254858

[24] Keuning BE Kaufmann T Wiersema R Granholm A Pettila V Moller MH. Mortality prediction models in the adult critically ill: a scoping review. Acta Anaesthesiol Scand. (2019) 64:424–42. 10.1111/aas.13527 31828760

[25] Lelubre C Vincent JL. Mechanisms and treatment of organ failure in sepsis. Nat Rev Nephrol. (2018) 14:417–27. 10.1038/s41581-018-0005-7 29691495

[26] Greco E Lupia E Bosco O Vizio B Montrucchio G. Platelets and multi-organ failure in sepsis. Int J Mol Sci. (2017) 18:2200. 10.3390/ijms18102200 29053592 PMC5666881

[27] Varela ML Mogildea M Moreno I Lopes A. Acute inflammation and metabolism. Inflammation. (2018) 41:1115–27. 10.1007/s10753-018-0739-1 29404872

[28] Yokouchi Y Oharaseki T Enomoto Y Sato W Imanaka-Yoshida K Takahashi K. Expression of tenascin C in cardiovascular lesions of Kawasaki disease. Cardiovasc Pathol. (2019) 38:25–30. 10.1016/j.carpath.2018.10.005 30419479

[29] Hasegawa M Nakoshi Y Muraki M Sudo A Kinoshita N Yoshida T. Expression of large tenascin-C splice variants in synovial fluid of patients with rheumatoid arthritis. J Orthop Res. (2007) 25:563–8. 10.1002/jor.20366 17262825

[30] Tucker RP Chiquet-Ehrismann R. The regulation of tenascin expression by tissue microenvironments. Biochim Biophys Acta. (2009) 1793:888–92. 10.1016/j.bbamcr.2008.12.012 19162090

[31] Page TH Charles PJ Piccinini AM Nicolaidou V Taylor PC Midwood KS. Raised circulating tenascin-C in rheumatoid arthritis. Arthritis Res Ther. (2012) 14:R260. 10.1186/ar4105 23193984 PMC3674624

[32] Shukla A Gaur P Aggarwal A. Tenascin-C levels, a toll-like receptor 4 ligand, in enthesitis-related arthritis category of juvenile idiopathic arthritis: a cross-sectional and longitudinal study. J Rheumatol. (2015) 42:891–6. 10.3899/jrheum.141365 25774061

[33] Gupta L Bhattacharya S Aggarwal A. Tenascin-C, a biomarker of disease activity in early ankylosing spondylitis. Clin Rheumatol. (2018) 37:1401–5. 10.1007/s10067-017-3938-5 29313272

[34] Zavada J Uher M Svobodova R Olejarova M Husakova M Ciferska H. Serum tenascin-C discriminates patients with active SLE from inactive patients and healthy controls and predicts the need to escalate immunosuppressive therapy: a cohort study. Arthritis Res Ther. (2015) 17:341. 10.1186/s13075-015-0862-4 26608564 PMC4660660

[35] Ulusoy S Ozkan G Mentese A Guvercin B Caner Karahan S Yavuz A. A new predictor of mortality in hemodialysis patients; Tenascin-C. Life Sci. (2015) 141:54–60. 10.1016/j.lfs.2015.09.011 26390818

[36] Sato A Aonuma K Imanaka-Yoshida K Yoshida T Isobe M Kawase D. Serum tenascin-C might be a novel predictor of left ventricular remodeling and prognosis after acute myocardial infarction. J Am Coll Cardiol. (2006) 47:2319–25. 10.1016/j.jacc.2006.03.033 16750702

[37] Yao HC Han QF Zhao AP Yao DK Wang LX. Prognostic values of serum tenascin-C in patients with ischaemic heart disease and heart failure. Heart Lung Circ. (2013) 22:184–7. 10.1016/j.hlc.2012.10.005 23177647

[38] Guo T Zhou X Zhu A Peng W Zhong Y Chai X. The role of serum tenascin-C in predicting in-hospital death in acute aortic dissection. Int Heart J. (2019) 60:919–23. 10.1536/ihj.18-462 31257330

[39] Celik A. The relationship between tenascin-C levels and the complexity of coronary lesion after myocardial infarction. J Atheroscler Thromb. (2011) 18:693–7. 10.5551/jat.6577 21512282

[40] Sato A Hiroe M Akiyama D Hikita H Nozato T Hoshi T. Prognostic value of serum tenascin-C levels on long-term outcome after acute myocardial infarction. J Card Fail. (2012) 18:480–6. 10.1016/j.cardfail.2012.02.009 22633306

[41] Yuan W Zhang W Yang X Zhou L Hanghua Z Xu K. Clinical significance and prognosis of serum tenascin-C in patients with sepsis. BMC Anesthesiol. (2018) 18:170. 10.1186/s12871-018-0634-1 30442110 PMC6238343

[42] Nozato T Sato A Hikita H Takahashi A Imanaka-Yoshida K Yoshida T. Impact of serum tenascin-C on the aortic healing process during the chronic stage of type B acute aortic dissection. Int J Cardiol. (2015) 191:97–9. 10.1016/j.ijcard.2015.05.009 25965612

