# Supplementary material for: Elevated Serum Tenascin-C Predicts Mortality in Critically Ill Patients With Multiple Organ Dysfunction
Source: Front Med (Lausanne). 2021 Nov 26;8:759273. doi: 10.3389/fmed.2021.759273 (PMC8661593; doi:10.3389/fmed.2021.759273)
Supplement: Supplementary file 1 [file Data_Sheet_1.PDF]

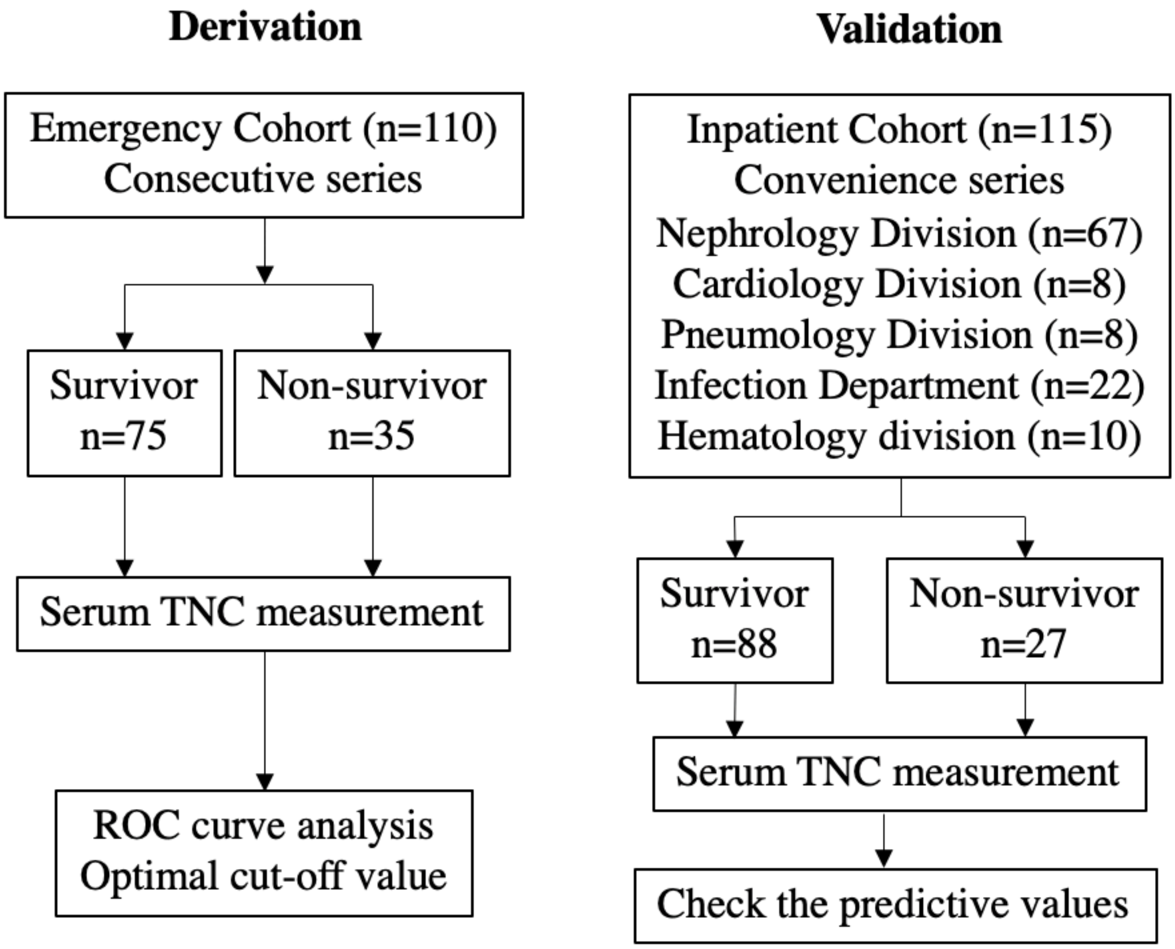

Supplement 1. Study design. The emergency cohort was a consecutive series. It was used to examine the value of serum TNC for predicting all-cause 28-day mortality by ROC curve. Serum TNC was detected within the 24 hours after enrollment. Optimal cut-off value was determined by Youden index. The inpatient cohort was a convenience series. It was used to validate the predictive value of serum TNC.
