# Supplementary material for: Elevated Serum Tenascin-C Predicts Mortality in Critically Ill Patients With Multiple Organ Dysfunction
Source: Front Med (Lausanne). 2021 Nov 26;8:759273. doi: 10.3389/fmed.2021.759273 (PMC8661593; doi:10.3389/fmed.2021.759273)
Supplement: Supplementary file 2 [file Data_Sheet_2.PDF]

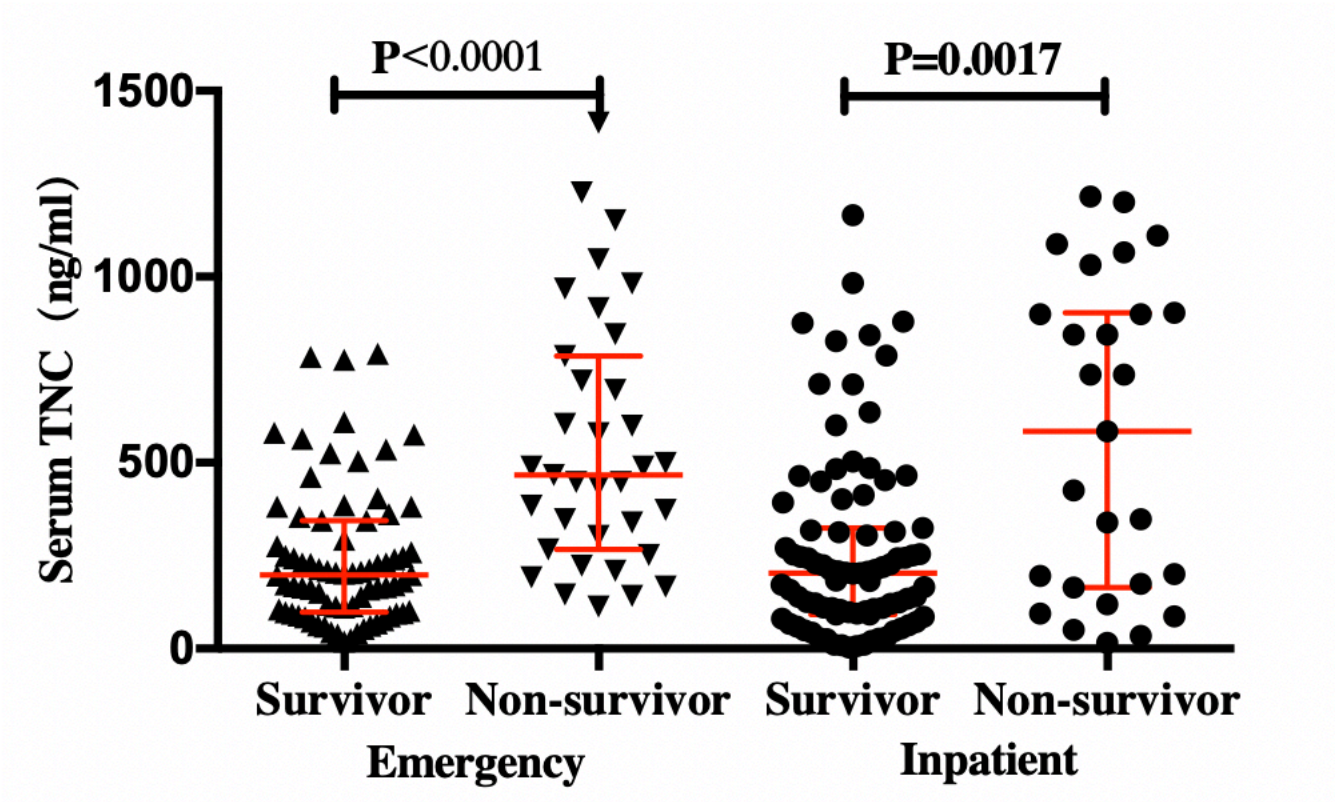

Supplement 2. Serum TNC in the survivors and non-survivors. Serum TNC in the non-survivors were significantly higher than that in the survivors in both emergency (derivation) cohort (467.0 (267.4, 786.3) vs. 197.6 (97.7, 343.8) ng/ml) and inpatient (validation) cohort (584.4 (164.4, 902.6) vs. 202.6 (91.4, 324.9) ng/ml).
