# Supplementary material for: Elevated Serum Tenascin-C Predicts Mortality in Critically Ill Patients With Multiple Organ Dysfunction
Source: Front Med (Lausanne). 2021 Nov 26;8:759273. doi: 10.3389/fmed.2021.759273 (PMC8661593; doi:10.3389/fmed.2021.759273)
Supplement: Supplementary file 3 [file Data_Sheet_3.PDF]

Supplement 3 The AUC of serum TNC and critical illness scores for all-cause 28-day mortality

	Emergency (Derivation) Cohort		Inpatient (Validation) Cohort	
	AUC (95%CI)	P	AUC (95%CI)	P
TNC	0.803 (0.717, 0.888)	< 0.001	0.745 (0.624, 0.865)	< 0.001
SOFA	0.808 (0.725, 0.891)	< 0.001	0.844 (0.776, 0.912)	< 0.001
APACHE II	0.762 (0.667, 0.857)	< 0.001	0.846 (0.780, 0.912)	< 0.001
SAPS II	0.779 (0.685, 0.872)	< 0.001	0.872 (0.808, 0.936)	< 0.001

AUC, area under receiver operating curve; CI, confidence index; TNC, tenascin-C; SOFA, Sequential Organ Failure Assessment; APACHE II, Acute Physiology and Chronic Health Evaluation II; SAPS II, Simplified Acute Physiology Score II.
